# Socio-digital stratification and the threshold effects of digital inclusion on maternal health access in Somalia

**DOI:** 10.1080/16549716.2026.2694167

**Published:** 2026-06-25

**Authors:** Abdirizak Mohamed Moumin, Abdalle Ali Yusuf, Khadar Mowlid Abdi, Abdirashid M. Yousuf

**Affiliations:** a Faculty of Business and Economics, Amoud University, Borama, Somalia; b School of Postgraduate Studies and Research, Amoud University, Borama, Somalia; c Research and Innovation Center, Amoud University, Borama, Somalia

**Keywords:** Antenatal care, mobile money, technology access, healthcare seeking behavior, Sub-Saharan Africa

## Abstract

**Background:**

Maternal health utilization in fragile states is hindered by complex social and structural barriers.

**Objective:**

This study examined socio-digital stratification and explored the threshold effects of digital inclusion on antenatal care (ANC) utilization in Somalia.

**Methods:**

Using data from the 2020 Somalia Demographic and Health Survey (*N* = 8,940), we constructed a Digital Inclusion Index (0–3) and employed survey-adjusted logistic regression models to estimate the odds of achieving four or more ANC visits (ANC 4+).

**Results:**

Only 8.4% of the women achieved the ANC 4+ threshold, with pronounced disparities between urban (14.0%) and rural/nomadic (5.5%) residents. Evidence consistent with a threshold effect was observed, whereby the initial transition from digital exclusion to basic connectivity (score = 1) was associated with the largest marginal gain in health seeking probability (OR = 1.55, *p* = 0.013). While mobile money penetration is high (85.2%), internet access remains deeply stratified by wealth (22.0% among the rich versus 1.0% among the poor). Interaction analysis suggests that digital inclusion complements but does not substitute for physical health infrastructure, partially mitigating distance-related barriers without eliminating the need for physical proximity to healthcare facilities.

**Conclusions:**

Digital inclusion is a critical entry point for improving maternal health access in Somalia. Policies should prioritize universal basic connectivity and SMS-based interventions, while simultaneously addressing persistent physical and socioeconomic inequities.

## PAPER CONTEXT

- **Main findings:** This study identifies a significant threshold effect where the initial transition to basic mobile connectivity provides the largest marginal gain in maternal health service utilization.
- **Added knowledge:** While mobile financial services have achieved broad penetration, a deep data divide persists, and digital tools function as complements to, rather than substitutes for, physical health infrastructure.
- **Global health impact for policy and action:** Public health interventions in fragile states should prioritize universal basic connectivity and text-based communication over data-intensive applications to avoid widening health inequalities.

## Background

Maternal health outcomes are fundamentally recognized as products of a complex interplay between social and structural determinants [1]. In low- and middle-income countries (LMICs), these determinants dictate the conditions under which women access essential care [2]. Sociodemographic factors, specifically the level of education and household income, remain the most consistent predictors of maternal service utilization [3]. For women in low-income African settings, these factors are often accompanied by severe transportation barriers [4]. In countries such as Nigeria, socioeconomic inequalities directly influence the stratification of healthcare delivery channels [5]. Similar patterns are observed in rural India, where illiteracy and geographical isolation create significant differences in care seeking [6].

The emergence of ‘Digital Determinants of Health’ has added a new dimension to this stratification, defining how technological access impacts health outcomes [7]. This ‘digital divide’ acts as a persistent barrier, maintaining health disparities among underserved groups [8]. Disparities in healthcare are now exacerbated by digital exclusion, particularly as systems move toward digitized records in the age of COVID-19 [9]. Digital health literacy has become a critical sociodemographic marker shaped by an individual’s age and previous technological exposure [10]. While digital health technologies are expanding across Africa, their deployment must be inclusive to prevent the further marginalization of vulnerable women [11].

Sociodigital stratification is most visible in the uneven distribution of mobile technology ownership, in which digital access is often partitioned by existing wealth hierarchies and gendered power dynamics. Evidence from 15 countries indicates that women’s mobile phone ownership is a significant indicator of health empowerment [12]. Mobile phone expansion is also linked to the attainment of development goals [13]. However, research suggests that women’s individual access to phones often lags their general household access, creating internal stratification [14]. In Bangladesh, digital health access is influenced by a combination of mixed-method socioeconomic factors [15]. To bridge these gaps, digital programs in Sub-Saharan Africa must prioritize women’s participation in program design [16].

Central to this stratification is the ‘threshold effect,’ which posits that a specific level of digital inclusion, rather than a linear increase in technology use, can trigger a significant shift in healthcare utilization. Access to traditional media, when moderated by mobile phone use, creates pathways for improved engagement with maternal services [17]. Exposure to mass media has been shown to significantly increase the likelihood of seeking institutional care [18]. Digital health interventions are now being utilized to fulfill the promise of equity in maternity care on a multicountry scale [19]. Information technology tools have the potential to reduce racial and socioeconomic disparities in maternal morbidity [20]. However, digitization can inadvertently exacerbate the health gap if the mothers’ perspectives are not integrated into the system [21].

Vulnerable pregnant women can benefit from targeted maternity applications such as MyCare [22]. The modern digital environment also requires health professionals and midwives to adapt to new roles in care delivery [23]. Digital birth equity interventions are increasingly being explored for their ability to reach resource-limited populations [24]. Nevertheless, the insufficient representation of the most impoverished women in population health datasets remains a major hurdle for future research [25]. For socially disadvantaged women, the perinatal period is often defined by specific facilitators that require culturally sensitive provider-side characteristics [26].

In the Somali context, the pursuit of sustainable health outcomes is challenged by a fragmented and privatized system [27]. Recent security challenges have further destabilized healthcare infrastructure [28]. Social determinants in fragile situations such as distance and lack of infrastructure remain the most significant hurdles to care [29]. This is exemplified by an ‘Infrastructure Paradox’ where the lack of formal banking and grid electricity has driven a necessity-based reliance on mobile-centric ecosystems, particularly among nomadic and rural populations [30]. Poverty remains a key driver of health exclusion, forcing choices that lead to suboptimal access [31]. For pastoralist women, wealth stratification is the primary barrier to institutional maternal service use [32]. In Mogadishu, women often feel powerless because of a lack of autonomy and reproductive health communication [33]. Mortality factors in such regions are deeply embedded in structural inequalities [34]. Even when government benefits are available, socioeconomic inequity often prevents marginalized populations from accessing health benefits [35]. The complex interplay between social determinants is a key contributor to maternal and infant mortality rates [36]. Expert consultations suggest that solutions must prioritize community-based health workers and sociocultural determinants [37]. Finally, chronic fragility leads to suboptimal access and persistently high maternal morbidity [38].

Despite the proliferation of digital health literature, there remains a critical knowledge gap regarding the interaction between digital inclusion and physical infrastructure in fragile states. Specifically, it is unclear whether technology acts as a substitute for or complements physical health infrastructure in environments where distance is a primary barrier. This study aimed to address this gap by analyzing the socio-digital stratification of maternal health utilization in Somalia. By examining the interplay between wealth, education, and digital access, this study sought to identify the threshold effects of digital inclusion and determine how technology-mediated connectivity influences the probability of achieving essential maternal health milestones in a high-barrier environment.

**Figure 1.**
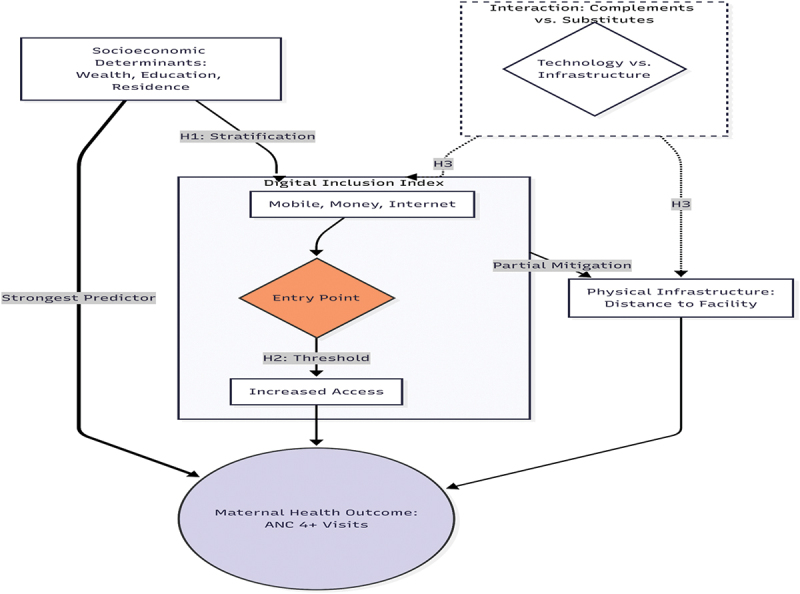
Conceptual framework of socio-digital stratification and maternal health access. The model illustrates the drivers of digital positioning (H1), the non-linear threshold effect of the digital entry point (H2), and the interaction between technology and physical infrastructure (H3) as complements in health-seeking behavior.

To systematically explore these dynamics, this study is guided by a conceptual framework (Figure 1) that maps how foundational socioeconomic determinants – specifically wealth, education, and residence – drive ‘socio-digital stratification,’ which in turn dictates a woman’s position on the Digital Inclusion Index. In this framework, Socioeconomic Determinants are defined as the structural inequalities that partition digital access, while Digital Inclusion represents a cumulative measure of an individual’s technological engagement (from basic mobile ownership to internet usage). At the core of the model is the Digital Entry Point, which hypothesizes a ‘threshold effect’ where basic connectivity yields the highest marginal gain in health-seeking probability, while higher scores result in diminishing returns. Furthermore, applying a ‘complements versus substitutes’ theoretical lens, this framework tests the interaction between Technology (measured by the Digital Inclusion Index) and Physical Infrastructure (measured by the perceived distance to a health facility). We hypothesize the following: (H1) Socioeconomic determinants significantly drive socio-digital stratification in maternal health access; (H2) There is a threshold effect wherein basic digital inclusion yields the highest marginal gain in antenatal care utilization; and (H3) Technology acts as a complement to, rather than a substitute for, physical infrastructure, partially mitigating distance-related barriers without eliminating the need for physical proximity to healthcare facilities.

## Methods

### Study area

Somalia, covering an estimated 637,657 km^2^, is a country in the Horn of Africa that is characterized by predominantly plateaued and hilly terrain. With a coastline of 3,333 km along the Gulf of Aden in the north and the Indian Ocean in the east and south, it has the longest coastline in Africa. It shares borders with Ethiopia in the west, Kenya in the southwest, and Djibouti in the northwest. Home to approximately 17 million people, the country is predominantly inhabited by ethnic Somalis. Mogadishu serves as the capital and acts as Somalia’s political and economic hub. The country’s arid and semi-arid climate combined with seasonal rainfall shapes its agriculture- and livestock-based economy. As a result, Somalia faces substantial health challenges, including the highest maternal mortality rates globally and significant barriers to healthcare access. In Somalia, the healthcare system is characterized by fragmentation and heavy dependence on private providers, humanitarian organizations, and non-governmental groups to deliver critical maternal and reproductive health services [2,27,31].

### Study design and data source

This study employed a cross-sectional design using a secondary analysis of data obtained from the 2020 Somali Demographic and Health Survey (SDHS), which is the first nationally representative survey carried out by the Somalia National Bureau of Statistics from 2018 to 2019 [27,31]. In addition to providing vital indicators for the entire nation, the study also targeted specific urban, rural, and nomadic areas, as well as each of the pre-war geographical districts. Because of security concerns, portions of certain regions, such as Lower Shabelle and Middle Juba, were excluded from the survey frame. The sampling strategy comprised a three-stage stratified cluster sampling technique in urban and rural areas, with proportionate sampling of primary sampling units (PSU) and secondary sampling units (SSU). In the first phase, enumeration areas (EAs) were sampled from digitally constructed dwellings. In the second phase, household listings were completed and 30 families were chosen from each EA. In the third stage, ever-married women aged 12–49 years and never-married women aged 15–49 years were interviewed using household and maternal mortality questionnaires.

**Figure 2.**
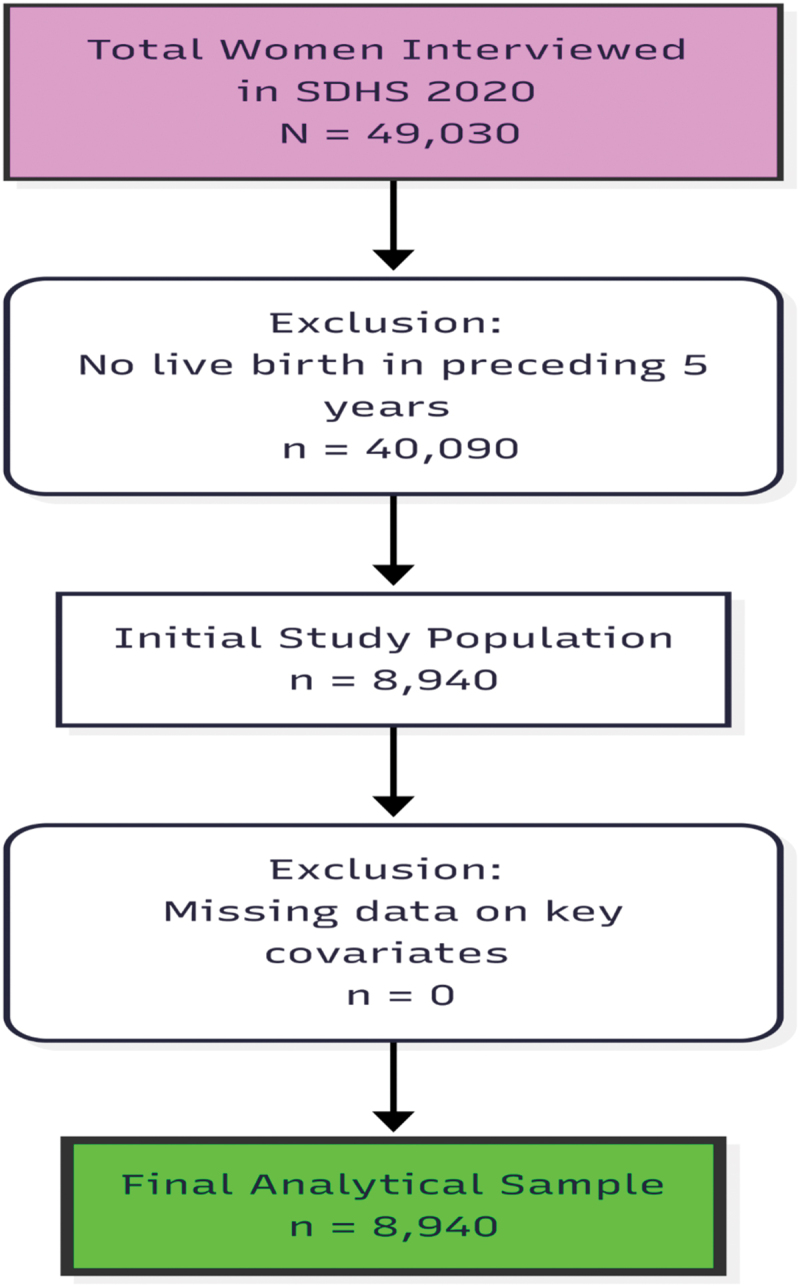
Flow diagram of the sample selection process for the study based on the 2020 Somalia demographic and health survey (SDHS).

For the purposes of this study, several exclusion criteria were applied to the initial dataset to derive the final analytical sample. Specifically, we excluded women who did not have a live birth in the five years preceding the survey, as well as any respondents with missing or incomplete data on our primary variables of interest (ANC visits and digital inclusion metrics). The sample selection and exclusion process are illustrated in the flow diagram in Figure 2. Because this study is a secondary analysis of a nationally representative survey, the sample size was not determined a priori. Instead, the final analytical sample consists of all eligible respondents meeting the inclusion criteria, totaling 8,940 women of reproductive age (15–49 years). This sample size provides robust statistical power (exceeding 80%) to detect small effect sizes in our multivariable logistic regression and allows for complex interaction modeling between digital inclusion and physical infrastructure barriers.

The survey instrument was administered through face-to-face interviews using structured questionnaires and a CSPro mobile data-collection application. Trained enumerators were responsible for data collection following rigorous training in interview techniques, sampling, and the recognition of maternal health indicators. The survey managed to capture information on nomadic populations through nomadic link workers and clan elders who facilitated linkages with mobile communities [33]. Ethical approval was obtained from the relevant institutional review boards of Somalia and the ICF Institutional Review Board.

### Variable operationalization

The dependent variable, maternal health utilization, was operationalized as a binary indicator for achieving the World Health Organization’s recommended threshold of four or more antenatal care (ANC 4+) visits. This variable was coded as 1 if the respondent reported four or more visits during her most recent pregnancy and 0 otherwise. The primary independent variable is the Digital Inclusion Index, a composite score ranging from 0 to 3, designed to measure ‘Socio-Digital Stratification.’ This index was constructed by summing three binary indicators: (1) individual ownership of a mobile telephone, (2) use of mobile phones for financial transactions (mobile money), and (3) use of the internet. This additive approach allows for the identification of ‘threshold effects,’ specifically testing whether the initial transition into the digital ecosystem yields disproportionate health gains.

### Covariates and structural barriers

Several socioeconomic and structural variables were included to control for confounding factors. Socioeconomic status was measured through the household wealth quintile (recategorized into Poor, Middle, and Rich), maternal educational attainment (no education, Primary, Secondary, and Higher), and maternal age. Geographic stratification was captured through place of residence (urban versus rural/nomadic). To address the ‘Complements versus Substitutes’ hypothesis, a structural infrastructure barrier was included: the respondent’s perception of the distance to a health facility. This was coded as a binary variable, where 1 indicates that distance is a ‘Big Problem’ and 0 indicates it is ‘Not a Problem.’ Additionally, traditional financial inclusion (ownership of a bank account) was included in the disaggregated model in contrast to mobile-based financial services.

### Statistical analysis

All analyses were performed using Stata version 16.0, and the results were adjusted for the complex survey design of the SDHS using the svy command suite. This adjustment accounts for the primary sampling units (clusters), stratification by region, and application of individual sampling weights to ensure national representativeness. The analysis was conducted in three stages:

1. **Descriptive Analysis**: Weighted descriptive statistics and bivariate correlations were calculated to map the landscape of the socio-digital stratification.
2. **Multivariate Modeling**: Survey-adjusted logistic regression models were used to determine the odds of achieving ANC 4+ visits. Model 1 disaggregated the digital components and included traditional banking to examine individual technological and financial impacts, whereas Model 2 used the composite Digital Inclusion Index to identify threshold effects. Average Marginal Effects (AME) were calculated to provide a substantive interpretation of probability shifts.
3. **Interaction Analysis**: An interaction term between the Digital Inclusion Index and the distance barrier was included to test whether technology acts as a substitute for physical proximity. The results of this interaction are presented as predictive margins and visualized through interaction plots to assess the ‘Complements versus Substitutes’ Framework.

### Ethical considerations

The study was conducted in accordance with the principles of the Declaration of Helsinki. The 2020 Somali Demographic and Health Survey (SDHS) protocol was reviewed and approved by the relevant institutional review boards of Somalia and the ICF Institutional Review Board. This research involved a secondary analysis of publicly available, de-identified, and anonymized data obtained via The DHS Program; therefore, further ethical clearance for this specific study was not required. Informed consent was obtained from all participants involved in the original survey by the primary data collection agency.

## Results

The analysis of maternal health access and socio-digital stratification proceeded in three stages. First, descriptive statistics were utilized to map the general landscape of digital inclusion, infrastructure barriers, and ANC utilization across rural and urban residences. Second, bivariate correlations were conducted to establish preliminary relationships between the study variables. Finally, survey-adjusted multivariable logistic regression and interaction analyses were employed to identify threshold effects and test whether digital inclusion acts as a complement or substitute for physical healthcare infrastructure.

### Sample characteristics and descriptive statistics

A descriptive analysis of the Somalia Health and Demographic and Health Survey (SDHS) revealed significant disparities in maternal health outcomes and digital access across residential lines Table 1. Only 8.40% of the total sample achieved the World Health Organization’s recommended minimum of four antenatal care (ANC 4+) visits. This figure is starkly bifurcated by geography; urban women are over 2.5 times more likely to reach this threshold (14.00%) than their rural and nomadic counterparts (5.50%). Infrastructure remains a primary hurdle, with 64.00% of respondents identifying distance to health facilities as a ‘big problem.’ This barrier was more pronounced in rural and nomadic areas (70.00%) than in urban centers (52.60%). The average Digital Inclusion Score (0–3) was 1.60 overall, though it was notably higher in urban areas (1.95) than in rural or nomadic areas (1.41). While mobile money usage is remarkably high across the sample (85.20%), internet penetration remains low at 9.30%, with a significant urban – rural gap (19.80% vs. 3.60%, respectively).

**Table 1.** Weighted descriptive statistics of maternal health and digital inclusion by residence.

Variable	Rural/Nomadic	Urban	Total
Health Outcome			
ANC 4+ Visits (Proportion)	0.06	0.14	0.08
Digital Inclusion			
Digital Inclusion Score (0–3)	1.41	1.95	1.60
Mobile Ownership (Proportion)	0.77	0.90	0.81
Mobile Money Usage (Proportion)	0.80	0.94	0.85
Internet Usage (Proportion)	0.04	0.20	0.09
Infrastructure Barriers			
Distance is a ‘Big Problem’ (Proportion)	0.70	0.53	0.64
Sample Size			
N	5,094	3,846	8,940

### The digital divide and socio-digital stratification

**Figure 3.**
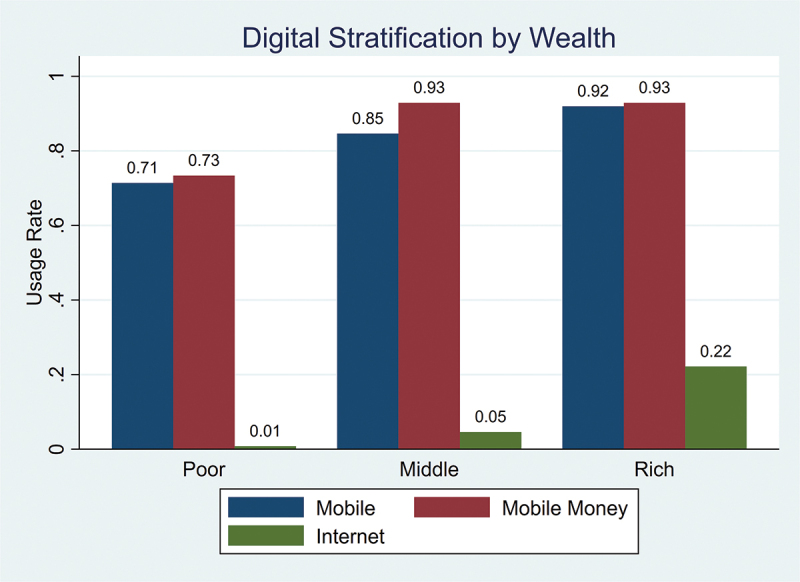
Digital stratification by wealth.

Figure 3 illustrates a clear pattern of ‘digital stratification’ based on household wealth. While mobile phone ownership and mobile money usage are relatively high, even among the poor (71.00% and 73.00%, respectively), Internet usage is almost non-existent in the lowest wealth quintiles (1.00%) compared to the rich (22.00%). This suggests that while basic mobile connectivity has achieved broad penetration, the ‘data divide’ remains a significant marker of socioeconomic status. Mobile money usage scales with wealth, reaching 93.00% of the wealthy, indicating that digital financial services are a near-ubiquitous tool for those with higher economic capital.

### Correlation analysis

Correlation matrix for Table 2 confirms that ANC 4+ visits were positively and significantly associated with the Digital Inclusion Index (*r*=*0.10*, p*<0.001*), urban residence (*r* = 0.13, *p* < 0.001), and wealth (*r* = 0.19, *p* < 0.001). Conversely, the infrastructure/distance barrier was negatively correlated with ANC 4+ visits (*r = −0.08, p < 0.001*), suggesting that physical proximity and digital connectivity are both critical, albeit distinct, determinants of health-seeking behavior.

**Table 2.** Bivariate correlations between study variables and ANC 4+ visits.

Variable	1	2	3	4	5	6
1. ANC 4+ Visits	—					
2. Digital Inclusion Index	.10***	—				
3. Age	−.01	.04***	—			
4. Urban Residence	.13***	.31***	.01	—		
5. Wealth Category	.19***	.43***	.05***	.52***	—	
6. Distance Barrier	−.08***	−.15***	−.02*	−.18***	−.20***	—

*Note. N* = 8,940. Pearson’s correlation coefficients are also reported. ANC 4+ = Four or more Antenatal Care visits. Urban Residence was a binary variable (1 = urban; 0 = rural/nomadic). The distance barrier is a binary variable (1 = Big Problem, 0 = Not a Problem). *p < .05. **p < .01. ***p < .001.

### Econometric analysis (logistic regression and marginal effects)

To evaluate the relationship between digital access and maternal health, we estimated the following two complementary logistic regression specifications Table 3. Model 1 disaggregates individual technologies, whereas Model 2 utilizes a composite Digital Inclusion Index to capture cumulative access. All estimates are reported as Odds Ratios (OR) relative to the baseline categories of no education, poorest wealth, rural/nomadic residence, no distance barrier, and a Digital Inclusion Score of 0.

**Table 3.** Logistic regression models predicting ANC 4+ visits (odds ratio).

*Predictor*	*Model 1: Individual Tech*		*Model 2: Digital Index*	
	OR	[95% CI]	OR	[95% CI]
Digital Inclusion				
Mobile Money Usage	0.75 (.07)	[0.55, 1.02]		
Internet Usage	1.18 (.29)	[0.87, 1.59]		
Bank Account	0.38*** (<.01)	[0.23, 0.62]		
Digital Index: 1			1.55* (.01)	[1.10, 2.19]
Digital Index: 2			1.16 (.31)	[0.87, 1.56]
Digital Index: 3			1.24 (.25)	[0.86, 1.78]
Socio-Economic Controls				
Age	1.00 (.71)	[0.99, 1.02]	1.01 (.45)	[0.99, 1.02]
Education: Primary	1.64* (.03)	[1.05, 2.55]	1.63* (.02)	[1.08, 2.47]
Education: Secondary	2.10** (.01)	[1.25, 3.52]	2.19** (<.01)	[1.34, 3.57]
Education: Higher	4.90*** (<.01)	[2.56, 9.39]	3.87*** (<.01)	[2.07, 7.22]
Wealth: Middle	3.30*** (<.01)	[2.03, 5.38]	3.00*** (<.01)	[1.89, 4.78]
Wealth: Rich	4.33*** (<.01)	[2.85, 6.56]	4.37*** (<.01)	[2.92, 6.54]
Urban Residence	1.22 (.44)	[0.73, 2.04]	1.27 (.39)	[0.74, 2.17]
Distance Barrier (Big)	0.86 (.32)	[0.64, 1.16]	0.93 (.60)	[0.69, 1.24]

*Note. N* = 8,940. OR = Odds Ratio; CI = Confidence Interval. Reference categories: Digital Index (0), education (none), wealth (poor), residence (rural/nomadic), and distance (Not a Problem). Model 1 examines specific technologies and Model 2 examines the composite Digital Inclusion Index. *p < .05. **p < .01. ***p < .001.

These results highlight a nuanced association between digital inclusion and maternal health. In Model 2, a Digital Inclusion Score of 1 was associated with a significantly higher likelihood of achieving ANC 4+ visits than was a score of 0 (OR = 1.55, *p* < 0.05). This indicates a threshold effect, whereby the transition from digital exclusion to basic inclusion yields the largest marginal gain in health seeking probability (Average Marginal Effect = 0.03, *p* = 0.02). The lack of statistical significance for the higher index scores suggests diminishing marginal returns on digital inclusion within the current Somali health infrastructure.

Socioeconomic control was the strongest predictors of ANC 4+ uptake. Women with higher education were significantly more likely to attend four ANC visits (OR = 3.87, *p* < 0.001) than those with no education. Wealth also exerts a powerful influence; being in the ‘Rich’ category increases the probability of ANC 4+ by 9.5% points (*p* < 0.001) relative to the ‘Poor’ category. Notably, traditional bank account ownership was strongly and negatively associated with ANC 4+ visits (OR = 0.38, *p* < 0.001). In the Somali context, this likely reflects institutional leapfrogging, where mobile-based financial systems superseded traditional banking as the primary infrastructure for the general population.

These associations are consistent with mechanisms, such as reduced transaction costs for seeking care, improved emergency communication with providers, and the use of mobile money to facilitate transport payments to distant facilities.

**Figure 4.**
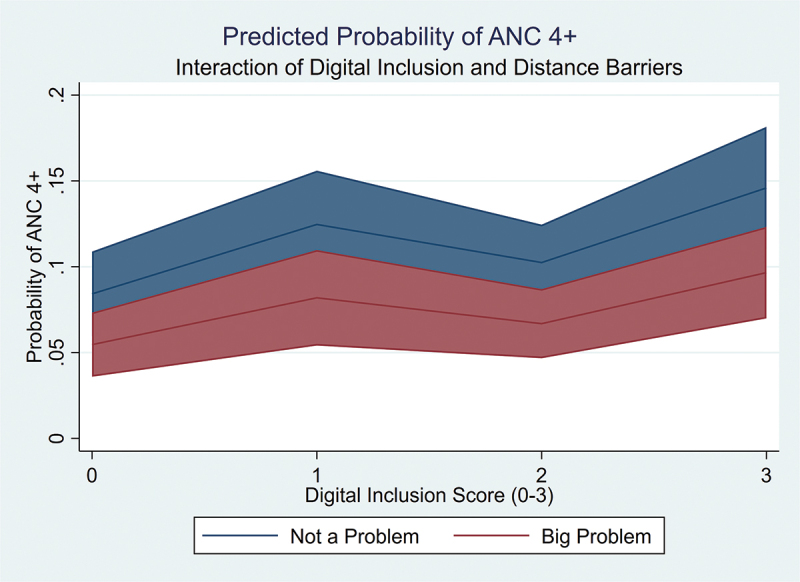
Predicted probability of ANC4+ interactions between digital and distance barriers.

Finally, Figure 4 illustrates the interaction between digital inclusion and distance barriers. In synthesis, the results of the econometric and interaction analyses confirm the study’s primary hypotheses. The data demonstrate a clear threshold effect, wherein the initial step from digital exclusion into basic digital connectivity provides the most significant marginal gains in maternal health access. Furthermore, the interaction analysis confirms that while digital tools partially mitigate spatial barriers for marginalized women, they function as complements to, rather than absolute substitutes for, physical healthcare infrastructure. While digital inclusion increases the predicted probability of ANC 4+ for all women, it does not fully close the gap created by physical infrastructure. Women who report that distance is ‘not a problem’ consistently maintain a higher probability of ANC 4+ across all levels of the digital index. This interaction pattern suggests that digital inclusion partially mitigates the disadvantages associated with distance but does not eliminate the structural importance of physical proximity to healthcare facilities; however, the persistent gap in predicted probabilities indicates that digital and physical infrastructure are complements rather than full substitutes in the production of maternal health. For women facing large distance problems, the initial transition into digital inclusion (Score 1) provides a visible boost in predicted probability (from 5.5% to 8.2%), suggesting that digital tools may partially mitigate the need for physical health infrastructure but cannot replace it.

## Discussion

The study identified a statistically significant ‘threshold effect’ at the point of digital entry, where the transition from total digital exclusion to basic connectivity (Score 1) yielded the most substantial marginal gain in ANC 4+ utilization (OR = 1.55, *p* = 0.01). This evidence suggests that the initial acquisition of a mobile device acts as a transformative ‘portable health link,’ providing marginalized and nomadic women with essential tools for service coordination and emergency communication. By reducing information and communication costs at this critical tipping point, basic connectivity facilitates institutional engagement in resource-limited settings, a finding that aligns with [17] on the impact of basic mobile access on institutional reach.

A persistent ‘second-level digital divide’ remains evident in Somalia, mirroring existing wealth hierarchies and complicating the path to health equity. While mobile money has achieved broad penetration, Internet usage remains a luxury of the rich (22%) compared with the poor (1%), indicating that the gap has shifted from hardware access to the depth of digital engagement. This stratification suggests that the poor remain excluded from the ‘digital determinants of health’ provided by Internet-based applications, supporting the argument by [9] that modern digital exclusion often exacerbates health disparities by restricting access to advanced digitized services.

The analysis further demonstrates that digital and physical infrastructure function as complements rather than substitutes in overcoming geographic barriers. Interaction analysis revealed that while digital inclusion increases the probability of ANC 4+ visits for women facing distance problems, a visible gap remains compared to those without such barriers. It is important to note that while distance was a major descriptive barrier, it was not a statistically significant independent predictor in the final multivariate model (*p* = 0.60). This suggests that the physical barrier of distance is heavily moderated by women’s financial and educational resources, confirming that technology cannot replace the clinical necessity of physical proximity to clinics or roads. These results challenge techno-optimist views by illustrating that digital tools are most effective when layered upon a functional physical healthcare infrastructure, which is consistent with the framework proposed by [4].

Somalia’s unique institutional landscape reveals the phenomenon of ‘institutional leapfrogging,’ where mobile-based financial systems have effectively superseded traditional banking. The surprising negative association between traditional bank ownership and health utilization (OR = 0.38, *p* < 0.001) likely reflects a selection effect, where formal banking is restricted to a tiny, specific elite whose health-seeking pathways differ from those of the general population. Conversely, mobile money serves as a critical liquidity tool for the majority to overcome immediate financial hurdles such as emergency transport. As noted by [27] in fragmented and privatized systems, digital financial inclusion via mobile platforms becomes a more relevant determinant of health access than traditional economic markers.

Despite the transformative potential of technology, traditional socioeconomic determinants, specifically education and wealth, remain primary predictors of maternal health utilization. Women with higher education were nearly four times more likely to achieve ANC 4+ visits (OR = 3.87, *p* < 0.001), underscoring that digital inclusion is mediated by users’ literacy and financial capacity to act on information. This confirms the perspective of [3] that socioeconomic status remains the ‘fundamental determinant,’ with digital tools serving as enhancers of, rather than replacements for, broader socioeconomic empowerment.

Based on these findings, public health policy in Somalia must prioritize ‘Universal Basic Connectivity’ by subsidizing mobile handsets and SIM cards for nomadic and low-income women as the initial entry point offers the highest return on health outcomes. To ensure inclusivity, digital health programs must remain ‘tech-agnostic,’ utilizing SMS and Interactive Voice Response (IVR) systems rather than data-heavy applications that exclude the 99% of poor women currently without internet access. Furthermore, the Somali health system should formally integrate with mobile money ecosystems to facilitate direct payments for maternal vouchers and emergency transport, leveraging the existing high penetration rates to remove liquidity barriers. Finally, policymakers must adopt a dual-track approach, ensuring that digital expansion does not replace investment in physical clinics and road networks, which are essential for clinical care.

While this study provides novel insights, it is important to acknowledge certain limitations. The cross-sectional design precludes the inference of strict causality between digital inclusion and health utilization. The reliance on self-reported SDHS data introduces potential recall bias, particularly regarding the number of ANC visits for births occurring up to five years prior. Additionally, the Digital Inclusion Index is a coarse measure that captures access, but does not account for the quality of use, data speeds, or specific digital health literacy. Furthermore, the models may not fully capture the influence of localized security volatility or clan-based social capital, which are critical latent variables shaping both physical safety and technological access in the Somali context. To build on these findings, future research should employ qualitative methodologies to unpack specific mediating pathways through which mobile connectivity facilitates care, such as logistical coordination or financial liquidity. It is also essential to investigate gendered power dynamics within households to determine whether women maintain full autonomy over digital devices and mobile money resources. Finally, longitudinal studies are needed to track the ‘digital evolution’ of maternal health behavior, specifically evaluating how the transition from basic SMS-based connectivity to broadband-enabled internet literacy impacts health outcomes over time in fragile states.

## Conclusions

This study establishes socio-digital stratification as a fundamental determinant of maternal health utilization in Somalia, identifying a statistically significant ‘threshold effect’ where basic mobile connectivity serves as a vital ‘portable health link’ for marginalized women. While technology offers a transformative entry point, its impact is moderated by deep-seated wealth and educational hierarchies, and it functions as a complement to rather than a substitute for physical infrastructure. Ultimately, achieving maternal health equity requires a dual-track strategy: the pursuit of universal basic digital connectivity must be paired with sustained investment in physical healthcare systems and road networks necessary for life-saving clinical care.

## Data Availability

The data that support the findings of this study are available from the Somalia National Bureau of Statistics (SNBS) microdata catalog at https://microdata.nbs.gov.so/index.php/catalog/50 upon reasonable request.

## Preprints

Not applicable. This manuscript has not been deposited in a preprint server.
